# Oxidative stress in cerebrovascular disease and associated diseases

**DOI:** 10.3389/fendo.2023.1124419

**Published:** 2023-02-17

**Authors:** Vijay Kumar, Kausik Bishayee, Soochul Park, Unjoo Lee, Jaebong Kim

**Affiliations:** ^1^ Department of Biochemistry, Institute of Cell Differentiation and Aging, College of Medicine, Hallym University, Chuncheon, Republic of Korea; ^2^ Biomedical Science Core-Facility, Soonchunhyang Institute of Medi-Bio Science, Soonchunhyang University, Cheonan, Republic of Korea; ^3^ Department of Biological Sciences, Sookmyung Women’s University, Seoul, Republic of Korea; ^4^ Department of Electrical Engineering, Hallym University, Chuncheon, Republic of Korea

**Keywords:** oxidative stress, neurodegeneration, stroke, risk factors, hypertension

## Abstract

Cellular aging is the most severe risk factor for neurodegenerative disease. Simultaneously, oxidative stress (OS) is a critical factor in the aging process, resulting from an imbalance between reactive oxygen and nitrogen species and the antioxidant defense system. Emerging evidence indicates that OS is a common cause of several age-related brain pathologies, including cerebrovascular diseases. Elevated OS disrupts endothelial functional ability by diminishing the bioavailability of nitric oxide (a vascular dilator), induces atherosclerosis, and impairs vasculature, which are all common characteristics of cerebrovascular disease. In this review, we summarize evidence supporting an active role of OS in cerebrovascular disease progression, focusing primarily on stroke pathogenesis. We briefly discuss hypertension, diabetes, heart disease, and genetic factors that are often linked to OS and are considered associated factors influencing stroke pathology. Finally, we discuss the current pharmaceutics/therapeutics available for treating several cerebrovascular diseases.

## 1 Introduction

Cerebrovascular diseases (CeVDs) are diverse and multifactorial diseases that include stenosis (stenosis of the carotid, vertebral stenosis, or intracranial stenosis) and aneurysms; these conditions generally result from the blockage (embolism) or rupture (hemorrhage) of blood vessels, ultimately leading to stroke (ischemic or hemorrhagic, respectively) (1–4). There are several well-known risk factors for stroke, including hypertension, obesity, high cholesterol, dyslipidemia, and diabetes; however, high levels of cellular reactive oxygen/nitrogen species (ROS/RNS) also contribute significantly to stroke/cardiovascular disease (CVD) pathogenesis. In healthy cells, ROS/RNS is maintained at a low concentration. It is important for normal cellular metabolism and contributes to normal physiological processes, including cell proliferation, differentiation, and apoptosis. However, elevated ROS/RNS, referred to as oxidative stress (OS), promotes many pathological conditions, including CVDs (2, 3). In the last decade, it has been repeatedly reported that increasing free radical activity plays an important role in the pathogenesis of vascular disorders, aging processes, and neurodegenerative diseases (5, 6).

As the brain is susceptible to OS, increasing amounts of ROS/RNS, impaired oxidants/antioxidant systems, and mitochondrial dysfunction together initiate neurodegenerative processes. In contrast, a lack of blood circulation (ischemia) to the brain (or any other region) limits the energy supply, which further elevates free radical concentrations and leads to neuron death (7). Additionally, a state of high OS induces several cellular and biochemical reactions, for example endothelial dysfunction, vasculitis (vascular inflammation), arterial remodeling, and blood–brain barrier impairment (8). Further consequences can include cerebral reperfusion injury, the blockage of cerebral blood flow, and cerebral bleeding or blood spillage. Considerable progress has been made in understanding brain functionality, and the importance of a balanced oxidant/antioxidant system for brain function is well-understood; however, how exactly elevated free radicals or OS cause CeVDs is ambiguous. Several studies have reported that ischemic stroke is a result of increased levels of cellular ROS, such as superoxide anions (O^2−^), hydrogen peroxide (H_2_O_2_), and hydroxyl radicals (OH^−^) (9–13). Here, we review the evidence on the contributions of free radicals/OS to stroke pathology. Additionally, we briefly discuss other associated factors and their relationships with stroke. Lastly, we review currently available pharmaceutic/therapeutic agents used to treat stroke.

## 2 Epidemiology of stroke

**Figure 1:**
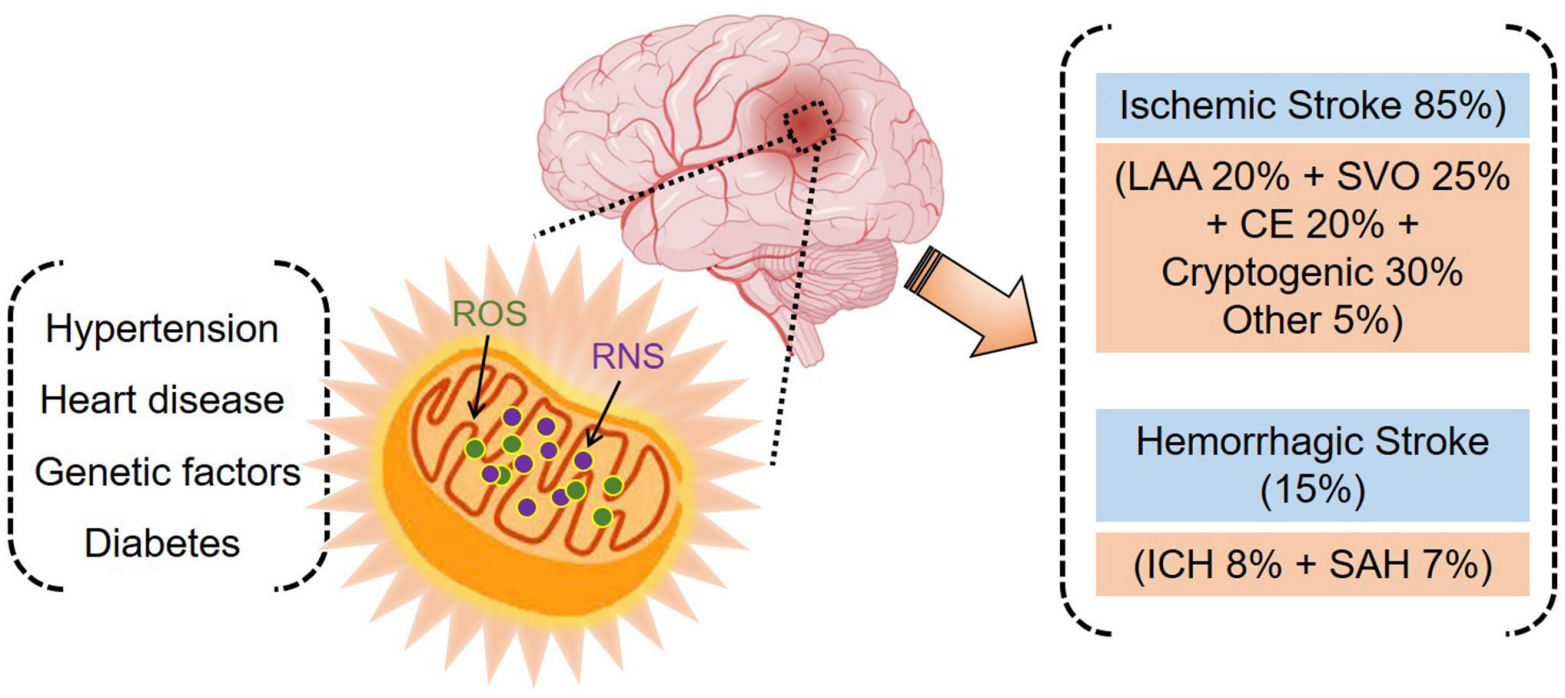
Stroke and associated risk factors. Oxidative stress plays a central role in stroke pathogenesis (ischemic and hemorrhagic) and is implicated in hypertension, heart disease, genetic factors, and diabetes.

On average, approximately 85% of strokes are ischemic and 15% are hemorrhagic. Ischemic strokes can be classified into large-artery atherosclerosis, small-vessel occlusion, cardioembolism, or cryptogenic ischemic stroke, among others. Hemorrhagic strokes can be classified into two groups: intracerebral hemorrhage or subarachnoid hemorrhage (**Figure 1**) (13, 14). CeVD has a severe impact on a patient’s quality of life, often resulting in long-term disability or death. For this reason, CeVD is listed as the second leading cause of death worldwide (15, 16). In 2019, 6.6 million deaths were caused by CeVD worldwide. Of these, 3.3 million were attributable to ischemic stroke, 2.9 million to intracerebral hemorrhage, and 0.4 million to subarachnoid hemorrhage (13). A previous study demonstrated that a total of 12.2 million stroke incidents and 6.55 million resulting deaths were recorded globally between 1990 and 1990 (17). In the United States alone, more than 795,000 people suffer stroke annually, resulting in a mortality rate of 48.6 deaths per 100,000 persons (18). According to an estimate (Centers for Disease Control and Prevention, U.S.A.), a 20.5% increase in the number of adults in the United States who will experience stroke is expected by 2030. A recent study demonstrated that type 2 diabetes mellitus and hypertension increase stroke occurrence by about 12.1% in patients with no stroke history. Moreover, recurrence of stroke within the 1 year follow-up period increased by 26.5% in patients with a history of stroke (19). High rates of ischemic stroke have been reported in North America, Southeast Asia, southern sub-Saharan Africa, North Africa, and the Middle East. Intracerebral hemorrhage shows a high prevalence in Oceania and Southeast Asia, while subarachnoid hemorrhage is prevalent in high-income regions of the Asian Pacific, North America, parts of Eastern Europe, and Oceania (13).

## 3 Oxidative stress in the progression of stroke

### 3.1 Oxidants and the antioxidant defense system

In a healthy brain cell, up to 1% of mitochondrial electron flow generates O_2_^−^, which is consumed by a variety of superoxide dismutases (SOD1, SOD2, and SOD3) to produce H_2_O_2_ molecules  (20). H_2_O_2 _is less toxic than O_2_^−^ but can react to generate even more dangerous compounds; for example, H_2_O_2_ can generate hydroxyl radicals (^−^OH) by reacting with Fenton’s reagent, and peroxynitrite anions (ONOO^–^) by reacting with nitric oxide (11). Several components of the antioxidant defense system, such as glutathione (non-enzymatic), thioredoxin, α-tocopherol (vitamin E), carotenoids, and ascorbic acid (vitamin C), interact with ROS to detoxify cells. Furthermore, the critical intracellular antioxidant enzymes glutathione peroxidase-1 and catalase convert H_2_O_2_ to water  (11, 21, 22). Failure of this antioxidant defense system is the leading cause of aging and neurodegeneration and has been reported in all types of stroke (23–26).

### 3.2 The compromised antioxidant defense system in stroke

Increased levels of OS (free radicals) in the brain lead to cerebrovascular and neurodegenerative diseases. The mechanism of OS elevation often results from a compromised antioxidant defense system. In the brain’s blood vessels, endothelial cells make up the innermost layer and are tightly surrounded by pericytes, followed by the basement membrane and astrocytic endfeet (27). Emerging evidence suggests that OS plays a central role in endothelial dysfunction and inflammation. The mechanism of ROS formation can be described as the blockage of cerebral blood flow that generates oxygen/glucose deficiency in neurons, with hypoxia ultimately triggering the decoupling of mitochondrial respiration (28, 29). This decoupling results in the production and accumulation of ROS and RNS products and the activation of xanthine oxidase. The increase in ROS/RNS products and ATP deficiency combine to initiate neurodegeneration and tissue oxygenation, leading to ischemia-reperfusion injury (28, 29). At the cellular level, OS can be measured by detecting the expression of crucial biomarkers (oxidative byproducts of different types of molecules); for example, DNA oxidation generates 8-hydroxydeoxyguanosine (8-OHdG), polyunsaturated fatty acid oxidation generates malondialdehyde (MDA), and lipid peroxidation generates 4-hydroxy-2-noneal (26, 30). Marker expression over a particular cellular concentration is generally estimated as an exalted or low state of OS. Several previous studies have demonstrated that patients with acute ischemic stroke express high levels of MDA (31–33). It is important to note, however, that MDA is not solely an OS marker; it is also a toxic molecule that can interact with DNA and protein and create mutagenic and atherogenic environments that can reduce cell survival (reviewed in (34)). Similarly, in a recent study, Liu Zhihua et al. established a relationship between high expression of the OS markers 8-OHdG and MDA and post-stroke cognitive impairment 1 month after acute ischemic stroke (35). The prolonged elevation of OS subsequently induces the accumulation of nitric oxide, an important vasodilator compound and a key player in many physiological processes (36), to a toxic level, causing inflammation and cytotoxicity and leading to mitochondrial dysfunction and neuronal death (25, 36). Additionally, previous studies have demonstrated that NADPH oxidase (NOX) plays a significant role in generating ROS in the brain. NOX gene deficiency or the inhibition of NOX activity significantly improves brain injury and neuronal function [reviewed in (37)].

In the antioxidant system, three SOD isoforms (SOD1, SOD2, and SOD3) scavenge ROS in different locations: SOD1 is cytoplasmic, SOD2 is located in the mitochondria, and SOD3 is extracellular. Combined, SOD1–3 establish the primary defense against ROS and functionally protect cells from free radicals and OS (38, 39). Recent studies indicate that SOD loss of function or genetic variation is associated with severe pathological diseases, including CVD and CeVDs (stroke) (10). In a previous study, six single-nucleotide polymorphisms (SNPs) within SOD genes were investigated to determine their association with ischemic stroke: rs17880487 and rs80265967 in SOD1, rs4880 and rs2842960 in SOD2, and rs2695232 and rs7655372 in SOD3. The SNP rs7655372 in the SOD3 gene was associated with an increased risk of ischemic stroke, and the SNP rs17880487 in the SOD1 gene was associated with an increased risk of cardiovascular mortality (10, 40). Furthermore, a mouse model with depletion of the key metabolic enzyme cystathionine beta-synthase or SOD1 developed hypertrophy without hypertension (41). Moreover, a cohort study demonstrated that deletion of 50 bp from the promoter region of SOD1 increased the risk of CVD and atherosclerotic heart disorder (42). Thus, taken together, these studies suggest that the absence or dysfunction of SOD1 disturbs the oxidant/antioxidant balance, leading to heart disease and CeVDs.

Similarly, Gpx-1, an intracellular antioxidant enzyme, reduces H_2_O_2_ to water and is implicated in neural cell viability in OS-induced neural degeneration. It has been reported that Gpx-1 knockout mice (Gpx-1^−/−^) exhibit a significant reduction in cerebral blood flow that induces susceptibility to cerebral injury and cerebral ischemia (43). However, it was previously reported that Gpx-1 knockout mice displayed a three-fold increase in infarct volume compared to normal mice. Moreover, pre-activation of the apoptosis marker, caspase 3, was reported in the stroke model, suggesting that the neuronal protective activity against the oxidative state and Gpx-1 deficiency may initiate the neuronal apoptosis program (43). This finding supports the importance of Gpx-1-mediated ROS scavenging, which is essential for neuronal viability, maintaining cerebral flow, and reducing microvascular perfusion that can lead to stroke.

It has been reported that oxidative/nitrosative stress is central to several neurodegenerative diseases (26). Gorg et al. (44) investigated the post-mortem cortical brain tissues of cirrhosis patients with or without hepatic encephalopathy (HE) and found that the HE brain expressed elevated levels of tyrosine-nitrated proteins, heat shock protein-27, and 8-hydroxyguanosine (a marker of RNA oxidation) with reduced activity of glutamine synthetase (44). This indicates that oxidative/nitrosative stress may be involved in HE pathogenesis, which is another risk factor for CeVDs. However, the authors did not discover an active association between oxidative/nitrosative stress and cirrhosis (44).

Additionally, hydrogen sulfide (H_2_S) is a gaseous signaling molecule that plays an important role in several physiological and pathophysiological processes and is a well-known essential protector against cellular OS (45). H_2_S is a marker of endothelial inflammation and dysfunction (reviewed in (46)), and its production in endothelial cells requires three key enzymes, cystathionine gamma-lyase (CSE), cystathionine beta-synthase, and 3-mercaptopyruvate sulfurtransferase. The CSE/H_2_S pathway has been reported to block atherosclerosis formation, and CSE-knockout mice exhibit severe atherosclerosis, supporting an anti-atherosclerosis role of H_2_S in the vascular system (45, 47). However, H_2_S also promotes antioxidant production by modifying the cysteine residues of upstream signaling molecules, including keap1/Nrf2, NFκB, and HIF-1α (45). Thus, H_2_S plays a dual role in the inhibition of atherosclerosis and induces the production of oxidant scavengers. Collectively, these findings indicate that compromised antioxidant defense systems or excessive ROS/RNS levels drive neuronal degeneration, which further leads to stroke or other brain-related diseases.

## 4 Oxidative stress and other factors associated with stroke

Risk factors often associated with CeVDs (stroke) are generally divided into two groups: lifestyle-related risk factors and health/medical risk factors. Lifestyle-related risk factors may include a lack of physical exercise, heavy drinking, obesity, and excessive use of drugs (such as cocaine). Health/medical risk factors include high blood pressure, high cholesterol, diabetes, CVDs, and a family history of CeVDs or genetic background. This section briefly summarizes the four common medical risk factors, in which OS is a common pathogenetic link.

### 4.1 Hypertension

High blood pressure can functionally or structurally affect the arteries of the body; this is known as hypertension. In a healthy adult, the normal blood pressure range is systolic ≤120 mmHg and diastolic ≤80 mmHg (120/80 mmHg). Hypertension is diagnosed when blood pressure increases to 130/90 mmHg (48). Hypertension is the number one risk factor for stroke, accounting for over 50% of ischemic and 70% of hemorrhagic stroke incidents, and is also a leading cause of dementia (35). Primarily, hypertension alters the structural and functional capabilities of cerebral blood vessels *via* hypertrophic and eutrophic remodeling. Hypertrophic remodeling triggers hypertrophy or hyperplasia of smooth muscles in an artery, leading to wall thickening and narrowing of the lumen space, further impairing essential blood supply (based on new guidelines from the American Heart Association, 2017) (41). In addition, hypertension initiates fibrinoid necrosis (lipohyalinosis) of the lenticulostriate arteries and causes intracerebral bleeding or hemorrhage (49). Increasing evidence supports the strong involvement of OS in hypertension and its deleterious effects on individuals’ health, as OS markers are elevated in hypertension (renovascular or malignant) and preeclampsia (12, 41, 49).

### 4.2 Diabetes

Diabetes mellitus (DM), a chronic metabolic disease caused by insulin resistance, leads to elevated blood glucose levels. An average of 20–40% of patients with diabetes have CeVDs, with asymptomatic cerebral atherosclerosis being among the most common (50). It has been reported that human patients and animal models of DM (type 1 or 2) exhibit accelerated atherosclerosis (51). The coronary plaque in DM patients contains high macrophage counts and larger necrotic core areas compared with non-DM subjects, which is similar to the high rate of calcification in DM patients (51). Similarly, these observations have been further validated in a mouse model that lacks the insulin receptor (−/−) in macrophages, which caused the progression of necrotic cores to advanced plaques (52). The larger size of necrotic cores reduces macrophage efferocytosis in obese (ob/ob) mouse models of DM (53) (see (51, 54) for targeted articles on the connections of diabetes in the pathogenesis of atherosclerosis). Thus, evidence suggests that DM leads to faster progression of atherosclerosis and promotes CeVD pathology.

### 4.3 Heart diseases

CVD is a major cause of death and a major risk factor for human health worldwide (55). Moreover, CVD has been a leading cause of death among noncommunicable diseases in the last two decades, causing 17.9 million deaths in 2019 (55). Several heart-related problems, such as atrial fibrillation, ischemic heart disease, cardioembolic infarction, vascular peripheral disease, chronic obstructive pulmonary disease, and atherothrombotic infarction, are strongly associated with stroke (or stroke subtypes) (56). This topic has been discussed in detail in (13, 39, 54–57).

### 4.4 Genetic factors

Identifying hereditary (genetic) factors underlying stroke pathology is essential to deciphering the molecular mechanism. In recent years, progress has been made with modern genotyping technologies. French et al. (58) postulated the involvement of the *PITX2* locus in stroke pathogenesis; indeed, *Pitx2* ^−/−^ mice exhibited a severe reduction in smooth muscle in cerebral vessels with increased vessel density. Similarly, the *ABO* locus has been associated with cardioembolic ischemic stroke (59). Several genetic factors have been identified that contribute to stroke risk at different levels, such as small-artery disease, large-artery atheroma, cardioembolism, hypertension, and dyslipidemia (60). For further reading, we direct readers to targeted articles in which known genetic factors have been classified and summarized (60, 61).

## 5 Diagnosis and treatment

The timely management of acute stroke after onset and selecting appropriate diagnostic/treatment methods are extremely important factors in stabilizing the patient. The accurate and timely recognition of stroke symptoms and first aid intervention can have a substantial impact on the outcome of the patient, potentially reducing mortality and stroke severity (62, 63). Several medical techniques have been developed to diagnose severe brain diseases, including stroke. For rapid clinical assessment, advanced brain scanning/imaging or neuroimaging tools are promising and required for precise and final confirmation of stroke after primary symptoms and blood analysis. Neuroimaging helps physicians develop timely treatment/surgery strategies. The most common neuroimaging technique is computed tomography (CT); CT scans can show subtle changes in cytotoxic edema, alterations in the gray-white matter, the hypoattenuation of basal ganglion, swelling, and blood vessel occlusion (64, 65). Advanced CT perfusion requires bolus administration (intravenous injection) to enhance the tissue visualization output. In addition to CT, magnetic resonance imaging or its more advanced version, magnetic resonance perfusion, are also commonly used; we direct readers to (66) for a review on this topic.

**Figure 2:**
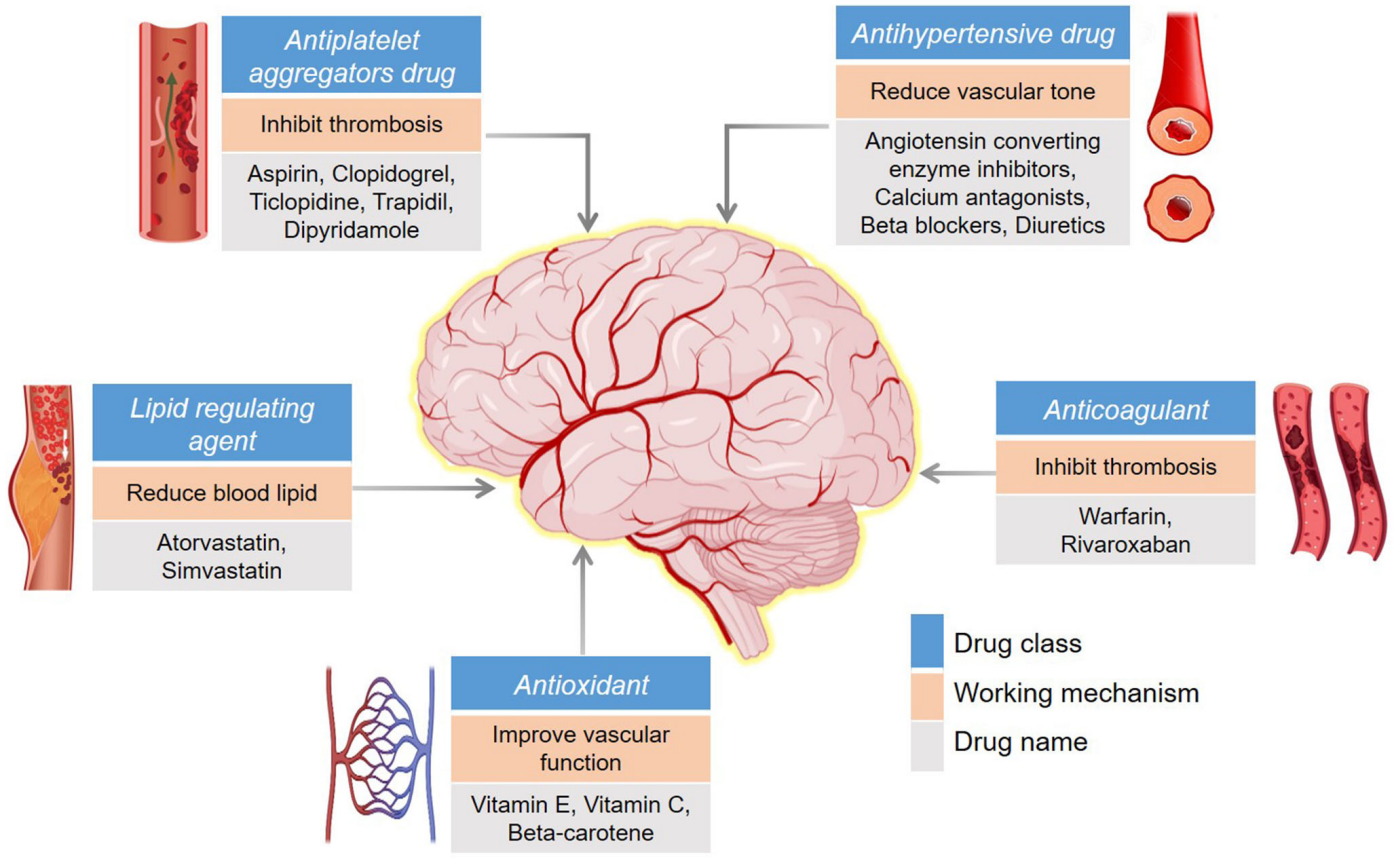
Common drugs for the prevention/treatment of stroke. The background color differentiates the drug class, working mechanism, and drug name as indicated.

Modern treatment modalities play an important role in the management and treatment of stroke. There are several strategies to prevent or cure stroke in patients. The most effective way to prevent CeVDs is to control associated diseases, including hypertension, diabetes, and heart disease  (67). Presently available drugs are classified into five groups based on molecular mechanisms and have overall effects on inhibiting vascular thrombosis, improving vascular function (or reducing tone), and lowering blood lipid concentration; some common drug names are shown in **Figure 2** (68). As most of these drugs mechanistically act on blood flow, they may have several side effects; for example, antiplatelet aggregation can cause severe bleeding problems  (69). Targeting free radicals by inhibiting their production is an effective strategy. The ideal targets are ROS-producing enzymes; for example, administration of allopurinol (metabolite; oxypurinol) to target xanthine oxidase and NS-398 and nimesulide to target cyclooxygenase2 (COX2) produced neuroprotective effects in transient and permanent ischemia (reviewed in  (70)). However, herbal medicines (derived from natural products) offer alternative stroke treatment or prevention options, typically with fewer side effects (71). Currently, a wide variety of herbs, including *Astragalus membranaceus, Angelica sinensis, Ligusticum chuanxiong, Paeonia lactiflora, Prunus persica*, and *Carthamus tinctorius*, may be effective against stroke and CeVDs as they exhibit solid antioxidant, anti-inflammatory, anti-apoptosis, and other neuroprotective properties (71–73). Bioactive compounds isolated from natural sources have been reported to reduce OS *via* many pathways. For example, leonurine (from *Leonotis leonurus*) and cornin (from *Cornus florida*) induced SOD1 and GPx activity, resulting in deceased ROS levels in a stroke model [reviewed in (74)]. Additionally, in a recent study, Deng et al. (75) investigated the neuroprotective activity of leonurine (an alkaloid compound) against the OS and nitric oxide/NOS pathways. PC12 cells treated with leonurine showed a significant reduction in OS (measured *via* MDA expression) and improved activity of the ROS scavenger enzymes SOD and GSH, indicating that leonurine may be a promising natural option for protecting neurons against OS.

## 6 Conclusions and prospects

The prevalence of CeVDs is increasing every year, including in younger populations, and thus demands urgent and effective treatment and prevention strategies. The occurrence of different metabolic diseases, CVDs, and hypertension significantly increases the life-long disability and death rates. Therefore, targeting a common cause may represent the most effective way to address this health issue worldwide. In the present review, we have discussed OS as a common root cause of pleotropic effects in several life-threatening illnesses, including stroke.

Significant progress has been made in understanding the mechanisms and molecular pathways that switch on ROS/RNS production or negatively regulate the antioxidant defense system. Over the past couple of decades, several mechanisms have been discovered to re-establish the antioxidant defense system and cellular homeostasis using different drugs or genetic manipulations. However, the exact molecular markers or diagnostic techniques used to detect mitochondrial dysfunction or OS at an early stage (before the onset of a severe condition) remain elusive. Further research efforts are necessary to develop accurate and effective treatment and prevention strategies for stroke and other diseases.

## Author contributions

VK: Original draft preparation. KB: Review and editing. SP, UL and JK: Supervision. All authors have read and agreed to the published version of the manuscript. All authors contributed to the article and approved the submitted version.
